# Micronodular lung lesions in an HIV positive patient – dilemmas in differential diagnosis

**DOI:** 10.1186/1471-2334-13-S1-P14

**Published:** 2013-12-16

**Authors:** Simona Claudia Cambrea, Ghiulendan Resul, Eugenia Basca, Elena Dantes, Stela Halichidis

**Affiliations:** 1 Clinical Infectious Diseases Hospital of Constanța, Romania; 2 Faculty of Medicine, “Ovidius” University, Constanta, Romania

## Background

In HIV positive patients from countries with a high burden of tuberculosis (TB), patients with lung cancer are often misdiagnosed as pulmonary tuberculosis, leading to delay in the correct diagnosis as well as exposure to inappropriate medication. Even though there are many similarities between the two diseases (involvement of the lung parenchyma and similar symptoms), there are also many differences between them like different etiologies, different consequences, and altogether different management.

## Case report

We performed a retrospective study on the hospitalizations and ambulatory records of an HIV positive patient. We present the case of a 61 year-old male patient, who was diagnosed with HIV infection in July 1996, acquired by heterosexual route. He used to be a smoker, 10 cigarettes/day, for the last 25 years, even though in the last 5 years he had quitted smoking. He was compliant for the HIV treatment, with a good evolution. In December 2010 he was diagnosed with pulmonary TB and he received specific treatment. After 4 months of antituberculous treatment the patient became asthenic, febrile, with productive cough, and weight lost. The radiologic (chest X-ray and CT) evolution was unfavorable. The suspicion of pulmonary neoplasm rose three weeks prior to his death. At the necropsy, macroscopic lung examination was very confusing between pulmonary TB and lung neoplasm, with a micronodular pattern similar with milliary TB. Histopathological examination evidenced a lung adenocarcinoma.

## Conclusion

A wrong or missed diagnosis of lung cancer by clinicians can lead to delays in treatment, wrong treatments, and finally death of the patient. In cases in which differential diagnosis is difficult to establish, histopathological examination establishes the final diagnosis.

